# First Selectively Imitate Responses, Then Associate With Function

**DOI:** 10.3389/fpsyg.2021.560653

**Published:** 2021-08-24

**Authors:** Elpida Tzafestas

**Affiliations:** ^1^Laboratory of Cognitive Science, Department of History and Philosophy of Science, National and Kapodistrian University of Athens, Athens, Greece; ^2^Department of Electrical and Computer Engineering, Technische Universität München, München, Germany

**Keywords:** imitation, association, selection, communication, social learning, behavioral prediction

## Introduction

Imitation is ubiquitous: human adults imitate, human infants, preschoolers, and adolescents all imitate, some animals too imitate. Moreover, imitation may be automatic, spontaneous, or deliberate and humans may specifically select their imitation models as well as the imitated target feature or object. Finally, language, gestures, motor patterns, and high-level behaviors can all be imitated at varying degrees of detail and in a variety of modulating contexts. These are some major aspects of the study of the broad class of cognitive processes called imitation, whether and how it has evolved and its relation to empathy, mind-reading, language, culture, and social learning in general (Hurley and Chater, 2005; Heyes et al., 2009; Jones, 2009, Shea, 2009; Claidière and Sperber, 2010; Gerrans, 2013; Hodges, 2014). Because of this ubiquity, imitation is sometimes used with diversified or more restricted meanings, such as mimicry and emulation (van Baaren et al., 2009; Whiten et al., 2009), and research efforts often center on the neural and behavioral organization that allows imitation to happen (Brass and Heyes, 2005).

We are focusing on the relation of imitation with association, as a basic mechanism of behavioral emergence, and with communication, as the general function of imitation (communication need not be thought as purely linguistic, though^1^). Our goal is to discuss the nexus of the three concepts (imitation, association, communicative function) and to propose a plausible view of the initial steps taken by evolution in the development of general social and communicative behavior. Thus, we propose an incremental process of generative and selective imitation starting from proto-imitation that replicates external signals without associating with target objects or functionality, and proceeding to proto-association that relates to higher-order imitation and attributes “meaning” or function to external signals. We are therefore discussing a model that allows proto-imitation outside explicit communication but may allow emergence of communication in the medium or long term ontogenetically. The idea is to separate response imitation from response association to external meaning so as to make room both for species that can imitate but not associate meaning and for disabled humans that cannot associate well or even proto-imitate well. Thus, we regard and model association as a generic functional concept, initially Hebbian at the neural level (“what fires together, wires together”), but more intricate at higher levels (Cooper et al., 2013).

In what follows, we present in order the proto-imitation concept and model, the predictions about its function in regular configurations or in cognitively extreme cases, the basic meaning association model and, lastly, further behavioral high-level predictions at organism level. We have verified computationally some of the predictions elsewhere and all of them match actual evidence obtained experimentally. We conclude with further thoughts about the proposed model.

## Selective Imitation

**Figure 1:**
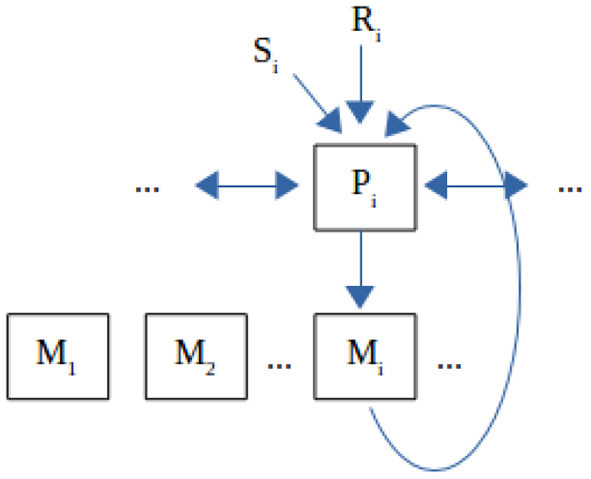
Functional organization of the imitation system. **(1) Affinity**. A signal S_i_ is initially perceived as a sensory pattern by a perceptual neural component P_i_ that elicits an originally random motor response M_j_, whose output can also be perceived by the same system. If responses that have a communicative value are selected by the environment (like for example a phonetic response from M_i_ matching the phonetic input S_i_, such as an “a” pronounced by self being perceived as an “a” pronounced by another) or if there appear P_i_'s that happen to match S_i_ and M_i_ output, then evolution will favor the emergence of the pathways of the type P_i_-M_i_, where the output of M_i_ is perceived by P_i_ as the closest to S_i_ among all M_j_. The pathway will show as higher population mass with larger total activation volume and diversity within the population, thus corresponding to our model of a “highly reproducing eigen-response.” **(2) Self-organization**. None of P_i_, M_i_ are born as invariants. All of them are subject to continuous reorganization, where P_i_ strives to better discriminate and assimilate (the closest) S_i_, M_i_ strives to co-stabilize with P_i_, etc. Moreover, there will be lateral interactions between P_i_'s and between M_i_'s because in principle P_i_'s overlap neurally to a degree, and so do M_i_'s. Thus, it takes some self-organization effort for each of them to stabilize to a discrete functional role in the system interfering the least with the other components. Again, with all these in place, evolution will favor the emergence of initial (“innate”) structures that can self-organize correctly and with little effort given consistent stimulation. Exploration is the process by which P_i_'s and M_i_'s change intrinsically. **(3) Higher-level association**. External signals are not just reproduced by the motor system, but they also refer to external objects (S_i_ refers to R_i_). Association of S_i_'s with R_i_'s will again pass through the organism's perceptual system (*via* a similar system as the previous level, but abstractly shown in the figure), so that either of them can finally trigger the same response M_i_. We note that this is a minimal mirroring property, but insufficient to produce “intention understanding” or anything cognitively more advanced. Structurally (meaning) association is a process parallel to the previous ones and the correct associations could emerge independently of whether the proper P_i_-M_i_ pathways have been built. But they are much easier to emerge when these pathways are in place, because then the S_i_-P_i_-M_i_ route may guide narrowing the scope of reference to the correct R_i_. **(4) Representations**. There is no such thing in this organization, because neither perceptual nor motor systems represent anything real. However, when an organism learns to act consistently on external input, it acts *as if* representations were present (but, we are aware that for some thinkers systematic consistency and an AS-IF representation IS a true representation; we accept this as valid common ground for communication between “computationalists” and “connectionists,” but see discussion by Chemero, 2009). **(5) Comments**. This model is similar to the proposal of Pickering and Garrod (2014) for language, except that unlike it there is selection instead of explicit forward modeling of M_i_ and that the overall functionality lies at the very primitive level, below the fully-fledged {semantics, syntax, phonology} configuration. Self-organization may be regarded as “intrinsic,” as proposed by Triesch (2013).

We are adopting the view that the ontegenetic development at the neural level follows the same principles as Darwinian evolution at the population level (Edelman, 1987) and that any novel responses should be generated internally and selected within the environment rather than be directly “instructed” by it. Thus, an organism can express a number of intrinsically encoded responses that can be produced spontaneously or automatically or unintentionally (cf. Heyes, 2011 and “reflex practicing and conditioning,” Piaget, 1947/2001) rather than computed rationally from the top to down (Gergely et al., 2002) and these can correspond loosely to real sensory or neural patterns such as vocal parameters. We call these responses “eigen-frequencies” or “eigen-responses,” on the one hand as an engineering term that shows a value that can excite the organism to produce a response, and on the other hand with an eye to a coupled neural oscillators or an entrainment implementation (Buzsáki, 2006; Ansermin et al., 2016). These responses can be modeled as dependent on a recognition or excitation threshold (T) and have accordingly varying degrees of affinity to a given signal. The response to an external signal is the eigenresponse with the highest affinity. At each step, new diversified eigenresponses emerge proportionally to the affinity of the previous ones. The highest matching responses reproduce massively, while the lowest ones vanish and are replaced by newly generated random eigenresponses. An exploration factor (E) is also necessary, which is the rate of random eigenresponse replacement independently of affinity. The overall affinity to an external signal is the average affinity of all the responses, thus it is internally generated and not externally imposed/designed/taught in any way. The internal evaluation through affinity makes the model selective rather than instructive, but we would gladly count shaping within a constant environment as indirect teaching. The affinity measure expresses *how “well” an organism recognizes and can reproduce (imitate) a signal* and may therefore serve as a basis for subsequent emergence of communication, when meaning is introduced to the interaction with the environment. The actual speed of imitation/learning and self-organization outcome in a given environment depend on both the organism's eigenresponse repertoire and setup and on the dynamics of stimulation by the environment. Figure 1 presents a general functional architecture that can support our model and how it makes sense from an evolution standpoint.

## Behavioral Extremes

Our model uses a number of cognitive parameters that have a “normal” range that we expect to have been tuned by biological evolution. We can predict that extreme setups will still be residually present in a population of organisms and will lead to extreme results that correspond to either behavioral deficits or exceptional performance. More specifically, if the set of eigenresponses is too small, the organism may not be able to imitate and develop the whole set of responses necessary for efficient long-term survival. Such an organism may appear as cognitively impaired and slow in learning or incapable of it. Similar results are predicted when the recognition thresholds (T) are too low, in which case spontaneous response matching will be rare to start with. A final complication is when the response exploration factor (E) is too low and the proper response cannot be reached or when, inversely, the factor is too high and the responses have a hard time stabilizing, because overwhelmingly many new responses are constantly appearing. A teacher could elicit the proper responses to be learnt, however, by using specialized and/or personalized schemes that teach progressively intermediate responses that are closer to the spontaneous response of the subject or with the aid of special social interaction schemes, such as games between “fast” and “slow” learners, where the former act spontaneously as additional teachers to the latter. A series of simple computational experiments have verified these predictions [Tzafestas, 2008, Tzafestas, (in preparation)]. Our predictions and results indicate that generative, non-goal-directed, proto-imitation may contribute to a number of phenomena involving successful social learning, as regularly expected, or social learning deficits, such as isolation of a slow learner that cannot learn all the necessary responses. In the context of communication, such a mechanism may precede the emergence of actual communicative function, rather than communication being the end to which imitation is the means.

## Association and Function

Next, the passage to *****true***** meaning association can be envisaged where originally meaningless matching responses are associated to external objects and dynamic associations are built and maintained (Catmur et al., 2009; Waxman and Gelman, 2009; García et al., 2014; Heyes, 2015,^2^; Sturdy and Nicoladis, 2017; Catmur and Heyes, 2019). The external references need not be pointed at or directly taught, although this might happen and can speed up learning (Eckerman and Stein, 1990; Ingersoll, 2010). Still, associations can be built spontaneously and reinforced in presence of multiple references in the environment, because statistically the correct reference for a given signal/response will be encountered much more frequently than other random references. We expect on average moderately rich environments in terms of wealth of stimuli to facilitate learning but cluttered ones to overstimulate and act as obstacles. Another prediction that was verified computationally (cf. above) is the emergence of multiple associations, in the sense of many responses associated with the same external reference, hence the basis for bilingualism and multilingualism. There are a number of additional intricacies concerning association. First, association has to be two-ways, from responses to references and from references to responses. Such associations can be developed either intrinsically in a Hebbian way and/or with the help of a reward-like contingency mechanism (Heyes, 2012; Cooper et al., 2013). In any case, how this could be implemented neurally would need to be worked out. Second, we need to carefully think the volume of associations that can be made. Birds, for example, can make a rather small number of associations, while the repertoire of a healthy human is comparatively enormous. Is big capacity a prerequisite for successful communication (for example, the capacity of the human vocal tract is indeed very big) or is it a drawback (because exploring a larger domain is harder)? It is also thinkable that reward/contingency mechanisms could be in the end necessary to learn a large set of responses or more complex responses, while purely Hebbian mechanisms could work for smaller sets or simpler responses.

## Other Predicted Behavioral Consequences

We can study accordingly extremes and deficits taken with the meaning/function association mechanism. First, reference salience is expected to be inversely proportional to the number of objects perceived in the environment and this can have drastic effects since extremely low salience would negatively affect the speed and ease of association and even block it altogether. Put otherwise, a subject that perceives too many things in the environment will be constantly distracted and hence slow or unable to learn, at least without special and personalized teaching. Such over-stimulation is thought sometimes to be the case in the autistic spectrum deficits (Remington and Fairney, 2017). Other predictions can be made as well. Bilinguals will be slower in learning concurrently their two first languages, but faster to learn the third. Because they are consistently stimulated twice as much and in a more complex manner than monolinguals, salience deficits will have less impact. Complete language replacement will be also hard, especially in the case of subjects with limited communicative repertoires.

All those predictions suggest that communicative and association deficits may sometimes arise even though the underlying imitation mechanism remains intact. We reiterate the case of the autistic spectrum disorders where social and communication deficits do not always go hand in hand with imitation problems and abnormalities (Leighton et al., 2008).

## Discussion

A number of assorted remarks can be made related to these ideas. First, association could be insufficient, but is it necessary in the first place? How else could meaning or function be assigned to external references provided the raw response imitation level exists? The alternative could be any top-down mechanism (Meltzoff and Prinz, 2002), such as innately given meanings waiting to be assigned (although again, some sort of limited association-like mechanism should be present for that) or an innate modular structure, refined at every step. We cannot rule out the possibility that some instances of such mechanisms exist in the human brain, however, as many authors argue, we think it is unlikely that they make the rule (Catmur et al., 2009; Jones, 2009; Froese and Leavens, 2014). Second, because we are interested in the behavioral predictions of our view, we are bypassing the sensorimotor correspondence problem and we assume that it is solved at the response imitation level in a basic associationist way. This would be an additional indication that selective mechanisms and especially association mechanisms appear at many places in the neural hierarchy.

Overall, we claim that a selective generative response imitation mechanism coupled with a higher level response association mechanism is capable of predicting many of the behavioral phenomena related to imitation in general, and especially a lot of abnormalities and deficits encountered in humans.

## Author Contributions

The author confirms being the sole contributor of this work and has approved it for publication.

## Conflict of Interest

The author declares that the research was conducted in the absence of any commercial or financial relationships that could be construed as a potential conflict of interest.

## Publisher's Note

All claims expressed in this article are solely those of the authors and do not necessarily represent those of their affiliated organizations, or those of the publisher, the editors and the reviewers. Any product that may be evaluated in this article, or claim that may be made by its manufacturer, is not guaranteed or endorsed by the publisher.

## Footnotes

^1^Imitation at any level has always a communicative value, in the sense that what is imitated really is or is expected to be functional in the environment, where function can be anything from immediate survival to social bond construction to human language learning. Like any other behavioral shaping mechanism, however, imitation may in the end fail to perform as expected.

^2^Heyes (2001, 2012, 2015) and Cooper et al. (2013) insist on the non-goal-directedness of imitation and on the sufficiency of fundamental association mechanisms (more intricate than Hebb-like) to solve the correspondence problem (Brass and Heyes, 2005), i.e., the matching of (sensory) stimulus to (motor) response. This is what we think that happens at the level of our response imitation level. Hence at all levels there are associative processes continuously at play, and the lower level processes enable the higher level ones that only receive consistent input when the lower levels have achieved relatively stabilized dynamics.
